# Experience with a Perfusion-Only Screening Protocol for Evaluation of Pulmonary Embolism During the COVID-19 Pandemic Surge

**DOI:** 10.2967/jnumed.121.262580

**Published:** 2022-04

**Authors:** Arun Kumar, Renée M. Moadel, Linda B. Haramati, Kenny Ye, Leonard M. Freeman, Lionel S. Zuckier

**Affiliations:** 1 Division of Nuclear Medicine, Montefiore Medical Center and Albert Einstein College of Medicine, Bronx, New York;; 2 Division of Cardiothoracic Imaging, Montefiore Medical Center and Albert Einstein College of Medicine, Bronx, New York;; 3 Departments of Radiology and Medicine, Montefiore Medical Center and Albert Einstein College of Medicine, Bronx, New York; and; 4 Division of Biostatistics, Department of Epidemiology and Population Health, Albert Einstein College of Medicine, Bronx, New York

**Keywords:** ventilation, pulmonary embolism, COVID-19, lung scintigraphy

## Abstract

The purpose of this study was to evaluate a pulmonary embolism (PE) perfusion-only screening (POS) protocol introduced during the coronavirus disease 2019 (COVID-19) pandemic surge. Subjects without dense parenchymal lung opacities were studied; those with less than 1 segmental perfusion defect were considered to have no PE, whereas those exhibiting 1 or more defects were indeterminate, mandating additional examinations to determine the final diagnosis. **Methods:** We analyzed demographic information, clinical data, imaging findings, and follow-up data from the electronic records of COVID-19 patients who underwent lung scintigraphy during the 60-d study period. **Results:** In total, 53 studies were performed on 17 COVID-19–positive and 36 COVID-19–negative patients. The POS protocol efficiently excluded PE in 79% of cases; the remaining 21%, indeterminate for PE, were generally referred for alternative testing or were directly anticoagulated. In patients with negative POS results, there was a very low mortality before hospital discharge (1/42) and normal results on follow-up studies (6/6). **Conclusion:** The POS protocol, implemented during the COVID-19 surge, efficiently and safely excluded PE in 79% of patients.

## Visual Abstract

**Figure:**
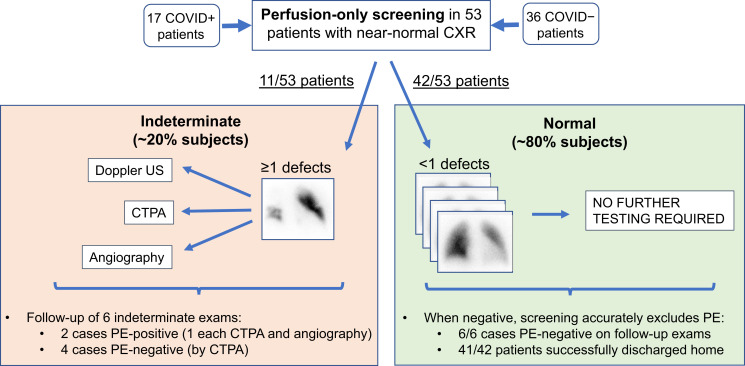

The purpose of this study was to evaluate a screening protocol for pulmonary embolism (PE) that we introduced during the initial coronavirus disease 2019 (COVID-19) surge (*1*) and was modeled on an algorithm used at our institution for evaluation of PE in pregnant women (*2*). No ventilation scintigraphy was performed (*1*). Absence of PE was based on detecting less than 1 segmental planar perfusion defect; studies demonstrating 1 or more segmental defects are considered indeterminate for PE, mandating additional examinations to determine the final diagnosis. Patients with known dense parenchymal lung opacities, in whom corresponding perfusion defects are anticipated, are directly referred for alternate studies such as CT pulmonary angiography (CTPA). We hope our experience will help inform the global discussion of best practices during periods of elevated risk from infectious respiratory pathogens.

## MATERIALS AND METHODS

Institutional review board approval was obtained with a waiver of the requirement for consent. We retrospectively retrieved demographic information, clinical data, imaging findings, and patient follow-up data from the electronic records of patients studied between March 21 and May 19, 2020, coincident with the initial surge of COVID-19 in our high-prevalence region. Continuous variables were summarized as mean ± SD, whereas highly skewed variables were described by median and interquartile ranges. The means of continuous variables were compared using the Welch *t* test unless otherwise indicated, and proportions were compared using the Fisher exact test. *P* values of less than 0.05 were considered statistically significant.

## RESULTS

### Demographic Findings

Fifty-three patients underwent lung scintigraphy during the 60-d period. Infection with severe acute respiratory syndrome coronavirus 2 virus was evaluated by polymerase chain reaction testing in 46 patients (13 of whom were positive and 33 negative), whereas 7 patients were categorized by clinical judgment (4 as infected and 3 as not). Demographic and laboratory values are listed in Table 1.

**TABLE 1:** Demographic and Clinical Findings

Parameter	All patients	COV+	COV−	*P*
COVID-19 status (*n*)				
Total	53	17	36	—
By polymerase chain reaction testing	46	13	33	—
By clinical assessment	7	4	3	—
Mean age ± SD (y)	49.0 ± 16.2	47.5 ± 17.2	49.8 ± 15.9	0.68
Females (*n*)	39 (74%)	10 (59)	29 (81)	0.11
Patients with serum creatinine ≥ 1.5 mg/dL (*n*)	19 (36%)	6 (35%)	13 (36%)	1
Patients with history of contrast allergy (*n*)	3 (6%)	1 (6%)	2 (6%)	1
Median D-dimer values (μg/mL)*	1.18 (IQR = 1.7)	1.34 (IQR = 2.3)	1.17 (IQR = 1.5)	0.66
Parenchymal findings on prior chest radiography (*n*)	13 (25%)	7 (41%)	6 (17%)	0.08
Patients with prior negative Doppler US (*n*)	8	2	6^†^	—
Patients with prior positive Doppler US (*n*)	2	0	2	—
Patients with prior nondiagnostic CTPA (*n*)	2	—	2^†^	—
Patients with ≥1 perfusion defects (*n*)	11 (21%)	3 (18%)	8 (22%)	1
Follow-up examinations on patients with ≥1 perfusion defects (*n*)	6/11 (55%)	0/3 (0%)	6/8 (75%)	0.06

*Reference value, ≤0.50 μg/mL. *P* values for D-dimer are based on Wilcoxon rank-sum testing.

^†^One patient had both negative Doppler findings and nondiagnostic CTPA.

IQR = interquartile range.

*P* values are for differences between COV+ and COV− subgroups.

### Prior Radiographic Findings

Chest radiographs or CT examinations were obtained for all patients within 0–5 d before the perfusion study (mean, 0.8 d). No dense parenchymal opacities were present; however, in 13 subjects there were 14 radiographic findings, consisting of ill-defined and nonsegmental opacities (7 patients), mild congestive heart failure (4 patients), and linear atelectatic changes (3 patients).

In the 17 COVID-19–positive (COV+) patients, 2 negative Doppler ultrasound studies of the legs were performed before scintigraphy. In the 36 COVID-19–negative (COV−) patients, there were 6 negative and 2 positive Doppler studies and 2 nondiagnostic CTPA examinations before scintigraphy.

### Scintigraphic Studies

Planar perfusion scintigraphy was performed according to Society of Nuclear Medicine and Molecular Imaging guidelines (*3*). After injection of 148 MBq of ^99m^Tc-macroaggregated albumin, planar images in 8 projections were acquired, at 180 s per view. Significant defects were wedge-shaped and pleura-based, with sizing conforming to Society of Nuclear Medicine and Molecular Imaging guidelines (*3*). Exceptions to the standard protocol occurred in 8 patients to whom reduced dosages of radiopharmaceutical were administered, 2 patients on whom low-dose ventilation was performed before scintigraphy, and 4 patients for whom SPECT/CT was additionally performed. Alterations in imaging confirmed but did not alter diagnoses based on planar perfusion imaging.

**FIGURE 1.**
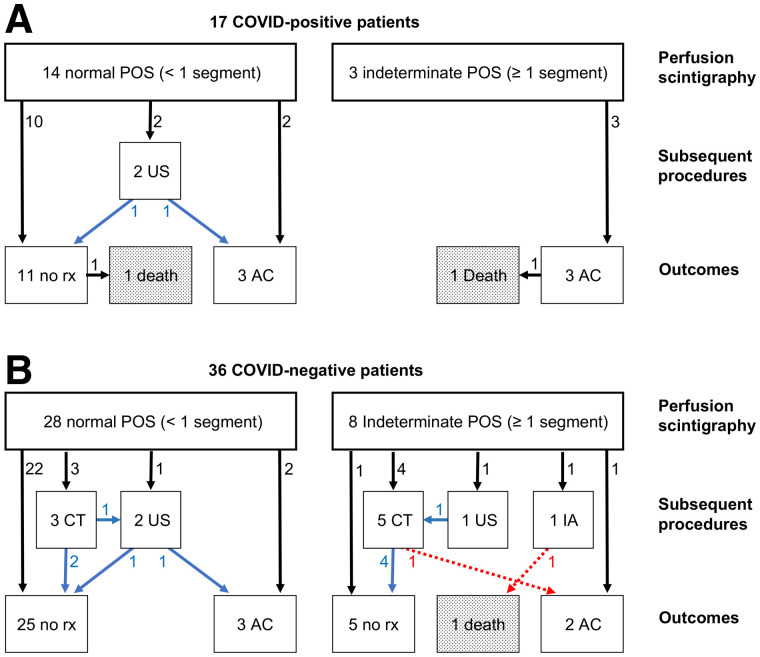
Diagnostic flowchart in 17 COV+ (A) and 36 COV− (B) patients. Arrows refer to flow of patients, whereas adjacent numbers indicate number of patients involved. Blue arrows indicate negative test result; red arrows signify positive test result. AC = anticoagulation; CT = CTPA; IA = interventional angiography; rx = therapy; US = leg Doppler ultrasound.

**FIGURE 2.**
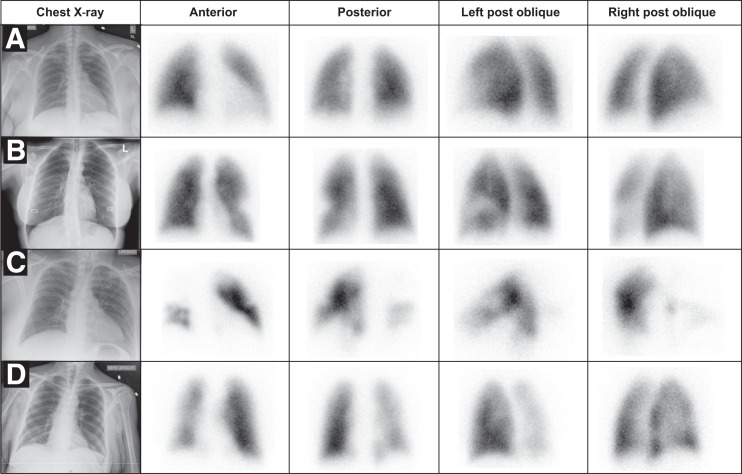
Key images of 4 representative patients. All chest radiographs demonstrate absence of significant opacities. (A) A 44-y-old woman, COV+ by polymerase chain reaction testing. No defects were noted on perfusion scintigraphy. Patient was not anticoagulated and was discharged without complication. (B) A 35-y-old woman, COV− by polymerase chain reaction testing. Well-defined segmental perfusion defect in superior lingula was indeterminate for PE. CTPA demonstrated normal pulmonary arterial perfusion; patient was discharged home without anticoagulation treatment. (C) A 43-y-old man, COV+ by polymerase chain reaction testing, with elevated D-dimer (19.7 μg/mL). Multiple bilateral segmental defects, especially involving right lung, were indeterminate for PE. Patient subsequently was discharged on anticoagulation treatment. (D) A 59-y-old woman, COV− by polymerase chain reaction testing. There is global decrease in perfusion of right lung, indeterminate for PE. CTPA demonstrated normal pulmonary arterial perfusion; patient was discharged home without anticoagulation treatment.

The frequency of segmental perfusion defects in the COV+ and COV− groups on planar perfusion scintigraphy was 18% and 22%, respectively (Figs. 1A and 1B); in 6 instances, single segmental defects were identified; in 4 patients, multiple segmental defects were identified; and in 1 patient there was a relative unilateral decrease in perfusion (Fig. 2).

### Clinical Follow-up in Patients with Normal Perfusion Results

Of 14 patients with normal perfusion in the COV+ group, 12 were not studied further whereas 2 underwent Doppler ultrasound of the legs, with negative findings in both cases (Fig. 1A). Three patients were placed on anticoagulation treatment, one of whom had atrial fibrillation. One of the 11 nonanticoagulated patients died of respiratory failure during hemodialysis 2 d after scintigraphy; the remaining 13 COV+ patients with normal perfusion were successfully discharged home.

Of 28 patients with normal perfusion studied in the COV− group, 24 were not studied further whereas 2 underwent CTPA, 1 underwent Doppler ultrasound of the legs, and 1 underwent CTPA followed by Doppler ultrasound, all with negative results (Fig. 1B). Three patients with normal perfusion in this group were anticoagulated, 1 with prior positive findings on leg Doppler ultrasound, 1 with a history of PE 4 y earlier, and 1 in atrial fibrillation. All 28 patients with normal perfusion in the COV− group were discharged home.

### Clinical Follow-up in Patients with Indeterminate Perfusion Studies

None of the 3 COV+ patients with perfusion defects received any follow-up examinations, and all were directly anticoagulated (Fig. 1A), one of whom died of respiratory failure a day after scintigraphy. Six of 8 COV− patients with indeterminate findings were referred for further diagnostic imaging: a catheter angiogram with positive results in 1, Doppler ultrasound of the legs with negative results followed by CTPA with positive results in 1, and CTPA examinations with negative results in 4 (Fig. 1B). Another COV− patient with indeterminate findings had prior positive Doppler results and was directly anticoagulated without additional imaging or an adverse outcome. The final patient had a perfusion defect described as atypical and was discharged home without therapy. The patient who underwent catheter angiography had a contraindication to heparin; thrombolysis was performed, with placement of an inferior vena cava filter. However, she died of multisystem failure 11 d afterward. The patient with positive CTPA results was anticoagulated, whereas the 4 patients with negative CTPA results were not. Of all 11 patients with indeterminate perfusion studies, 9 patients were therefore ultimately discharged home (2 COV+ and 7 COV−).

## DISCUSSION

A variety of approaches to performing lung scintigraphy were considered during the early COVID-19 period (*4*), designed to balance tension between potential spread of infection when ventilation scintigraphy is performed and suboptimal specificity of scintigraphy when ventilation is omitted. These considerations informed our approach, which used perfusion scintigraphy in a screening role, relying on the established sensitivity of perfusion scintigraphy to identify disease and not creating new criteria of interpretation.

The most salient observation regarding the perfusion-only screening (POS) protocol is that approximately 80% of patients had less than 1 segmental defect and required no further testing. By restricting the patients whom we studied to those with a relatively clear chest radiograph, we succeeded in obtaining a subgroup of subjects with a low prevalence of defects, thereby excluding PE efficiently and validating the anticipated benefit of the protocol. A subgroup of 13 patients was imaged despite the presence of mild parenchymal abnormalities, which did not interfere with performance of the examination.

The POS protocol was accurate and safe. Of 6 patients who underwent additional diagnostic examinations after negative results, no emboli or thrombi were confirmed. Among all 42 patients with negative POS results, there was only 1 fatality, a COV+ patient who died of respiratory failure. Of the 11 subjects with indeterminate perfusion scintigraphy, 6 underwent further testing, 2 of whom had PE substantiated on follow-up, demonstrating use of POS as a screening examination. One COV− patient with proven embolism and a contraindication to anticoagulation succumbed to multisystem failure, whereas a second COV+ patient, who was placed on anticoagulation treatment, died of respiratory failure 1 d after scintigraphy.

Limitations of our retrospective study include an inability to determine outcome by the optimal gold standard of 60 d of follow-up, because most patients were not enrolled within our health-care system after discharge. As well, we cannot easily reconstruct how many patients were referred away from scintigraphy because of radiographic abnormalities or other considerations and which alternative examinations they underwent. A final limitation relates to the generalizability of our findings. The prevalence of segmental defects in patients referred for testing is highly dependent on specific referral patterns and the regional incidence of disease. Nonetheless, the relatively robust results we obtained suggest that this protocol could be cautiously extrapolated to similar environments with ongoing monitoring of efficacy.

Although we imaged by planar scintigraphy, a similar screening protocol can be based on SPECT imaging, used in many regions (*5*). It is also possible that interim specificity can be improved by factoring in pretest probability (*6*) or by performing SPECT/CT, in which anatomic CT information can be used as a partial surrogate for ventilation (*6*–*8*).

An intriguing consideration deriving from our experience is whether performing a perfusion study first as a screening test should continue in noninfected patients with relatively clear chest radiographs in whom PE is being excluded (*9*). Only if segmental defects are noted on the perfusion study would a completion ventilation study or complementary imaging be performed.

## CONCLUSION

We have reviewed our experience during 60 d of the initial COVID-19 surge using a POS protocol implemented in 53 patients with minimal abnormalities on baseline chest radiography. This screening protocol efficiently and safely excluded PE in 79% of the patients studied.

## DISCLOSURE

No potential conflict of interest relevant to this article was reported.

## Notes

KEY POINTS **QUESTION:** Is the POS algorithm, introduced during the COVID-19 surge to assess for pulmonary embolus, an efficient and safe method of evaluation? **PERTINENT FINDINGS:** We retrospectively analyzed chart data from 57 patients studied over the initial 60 d of the COVID-19 surge. POS was able to efficiently exclude PE in 79% of patients and was accurate and safe as determined by follow-up imaging and hospital discharge. **IMPLICATIONS FOR PATIENT CARE:** POS protocols can safely be implemented when ventilation scintigraphy cannot be performed and serve to efficiently exclude PE in most patients.
